# The relationship between telework from home and the psychosocial work environment: a systematic review

**DOI:** 10.1007/s00420-022-01901-4

**Published:** 2022-07-13

**Authors:** Jolien Vleeshouwers, Lise Fløvik, Jan Olav Christensen, Håkon A. Johannessen, Live Bakke Finne, Benedicte Mohr, Ingrid Løken Jørgensen, Lars-Kristian Lunde

**Affiliations:** National Institute of Occupational Health, P.O. Box 5330 Majorstuen, 0304 Oslo, Norway

**Keywords:** Working from home, Telework, Telecommuting, Remote work, Psychosocial work environment

## Abstract

**Objective:**

Telework from home (TWFH) has become routine for many, yet research on how this may affect the psychosocial work environment is sparse. To understand the effects that TWFH may have on the psychosocial work environment, this systematic literature review identified, evaluated, and summarized findings on the association of TWFH with factors of the psychosocial work environment.

**Methods:**

Searches were conducted in MEDLINE, Embase, Amed, PsycINFO, and PubMed. The topic of the study reflected TWFH, and subjects should be office workers employed at a company. Outcomes should reflect psychosocial work environment factors. Inclusion criteria stated that studies should be primary, quantitative, and published in a peer-reviewed journal. English language publications dating from January 2010 to February 2021 were included. Risk of bias was assessed using the Newcastle–Ottawa scale (NOS) and quality of overall evidence using Grading of Recommendations Assessment, Development and Evaluation (GRADE).

**Results:**

Searches resulted in 3354 publications, and after screening rounds 43 peer-reviewed original studies satisfying predetermined inclusion and exclusion criteria were included. Fourteen individual psychosocial work environment outcome categories were studied. Limited overall evidence to support effects of TWFH on the included work environment outcomes, with evidence being rated either of low or very low quality. Flexibility and autonomy are discussed as potential mediating variables in the relationship between TWFH and the psychosocial work environment.

**Conclusion:**

There is a lack of high-quality research investigating effects of TWFH on the psychosocial work environment. To suggest TWFH guidelines or recommendations, there is a need for research with high-quality longitudinal designs, precise measures of time use and location of work, and validated measures of factors known to be of importance.

**PROSPERO registration number** CRD42021233796.

**Supplementary Information:**

The online version contains supplementary material available at 10.1007/s00420-022-01901-4.

## Introduction

With increased globalization, technological progress, and digitalization the previous decades, a large part of the international working life has been introduced to significant transitions. The new aspects introduced have altered both the content of work and how it is organized and carried out (Kingma 2019). One remarkable event is the introduction of telework solutions, which have been implemented since the 80s, when technological innovations made such distant working solutions a possibility (Shamir and Salomon 1985). For this type of work arrangements, different terminologies, such as remote working, telework, flex-work, and working from home, are often used interchangeably. However, important differences exist, both in nature of the physical location of the work, as well as in the implied flexibility and choice by the employee. Distinctively, telework would be a subcategory of remote work and can be defined as “the use of telecommunications technology to partially or completely replace the commute to and from work”, with the work site typically being an office building provided by employer. Telework from home (TWFH) would be a further specification, indicating that the telework is being performed from home (Mokhtarian 1991; International Labour Organization 2020). On the other hand, flexi-work or flexible work may reflect flexibility not only in workplace but also in when during the day to engage in work and the number of working hours (Hill et al. 2008).

From its introduction decades ago, telework has slowly become more widespread, and pre-COVID-19 around 15% of European workers engaged in some form of telework from their home, with the Nordic countries among those most frequently implementing telework adaptations (Eurofound and International Labour Office, 2017; European Agency for Safety and Health at Work, 2019; Messenger, 2019). After the COVID-19 pandemic hit, several measures were taken to reduce virus spread, and following there was a drastic increase in the occurrence of employees TWFH. In April 2020, over one-third (39%) of EU workers carried out their work remotely from home. By July, this percentage had increased to almost half of the working force (48%), with 34% doing their work exclusively from home and 14% working partially from home (Eurofound 2021a). When asked on preferences regarding TWFH after the COVID-19 pandemic, most workers would prefer to combine work from home with work from their employer’s premises in future (Eurofound 2021b).

The context in which we engage in work influences the experience we have of that work. The physical and psychosocial working conditions are patently different when comparing working from an office location to an employee’s home. For instance, a basic psychological need for humans is social connection (Ryan and Deci 2017), and a change from a physical to a virtual workplace could affect how and to which degree these needs are fulfilled. This change could also introduce a feeling of professional isolation, which may have a negative impact on how an employee perform his/her work (Mann et al. 2000; Vega 2003; Golden et al. 2008). Another basic need is the need to feel in control (autonomy) (Ryan and Deci 2017), a work environmental factor linked to work engagement, motivation, and work performance (Dieker et al. 2019; Muecke and Iseke 2019). Several studies have shown that increased digitalization and the introduction of new technologies may alter employees’ perceived feel of control over their own work situation (Christensen et al. 2020). Further, social support from leaders and colleagues has also shown to be of importance to the employee (Christensen et al. 2018; Finne et al. 2014). These are factors that may be related to social interactions, which could play out differently in a virtual working environment compared to a physical working environment. As such, our physical working location may influence not only concrete, observable, work environment outcomes, such as productivity, but also how we evaluate our psychosocial work environment, e.g., job demands, role clarity, control, support etc. (Sundstrom 1986). A recent report suggests that working at home may lead to changing working time patterns, where working time may be more irregular and unpredictable (Eurofound 2020). However, the same report suggests that this may lead to a flexibility in arranging work around family needs, resulting in a better work–life balance for some, while causing intensification and overload for others. This duality, digital working solutions and increasing flexibility and autonomy, yet causing a blurred boundary between work and free time, is referred to as the empowerment/enslavement paradox (Cohen et al. 2021; Jarvenpaa et al. 2005).

Working at home will in most cases mean that you perform work tasks using a telephone, computer, internet and other technological communication solutions, i.e., TWFH (International Labour Organization 2020). The compatibility between such an arrangement and the work performed will vary depending on the nature of the work tasks. Despite the fact that several occupational groups have had to adapt work tasks within the framework of TWFH in recent times due to a global pandemic, employees with office work make up the majority. Furthermore, outside of extraordinary times, TWFH while not self-employed will most likely apply almost exclusively to office workers. Therefore, the current review was limited to employed (i.e., not self-employed) office workers.

While the COVID-19 pandemic has resulted in increased TWFH, the circumstances in which most workers were expected to work remotely are likely to be different from TWFH under regular circumstances. Extraneous circumstances, such as the lack of flexibility or choice, possible lack of technological and ergonomically beneficial solutions at home due to the promptness of lockdown measures, and the fact that many had family and children at home at the same time, suggest that pandemic TWFH situations may not be representative of TWFH experiences post-pandemic, when these measures are discontinued. Furthermore, uncertainty, worries, dissatisfaction and fear experienced by many as a result of the pandemic itself may affect appraisals of the work situation as well. Therefore, the present review will not include studies that investigated TWFH during lockdown conditions.

With the rise of TWFH opportunities, research has focused on the possible work-related effects, both on the worker, and on the workplace and work environment, e.g., organizational culture and turnover intention. In this review, we exclusively investigate employees that have defined TWFH to increase relevance and reduce heterogeneity. The present systematic review aims to identify, evaluate, and summarize the findings of recent relevant studies investigating the associations between TWFH and the psychosocial working environment.

## Methods

The present systematic review is part of a larger research project initiated by the Norwegian National Institute of Occupational Health, aiming to investigate potential effects of TWFH on workers’ experience of the working environment, as well as their health. A combined search was initiated and the overarching project was protocol registered in the international register for systematic reviews, PROSPERO (PROSPERO ID # CRD42021233796), and follows PRISMA guidelines (Page et al. 2021).

### Search strategy and selection criteria

Since the current systematic review was part of a larger project spanning not only the work psychosocial environment as an outcome, but also employee health, the search strategy described below reflects both outcomes initially, then a narrower selection categorized by outcome (work environment effects vs health effects).

Two identical systematic searches were carried out in October 2020 and the second in February 2021. The second search was performed to capture any studies that had been published after the first. The searches scoped six databases: Scopus, PubMed, Medline, Embase, PsycInfo and Amed.

The topic of the study should reflect TWFH, subjects should be employed at a company (i.e., not self-employed), and the main work tasks should reflect office work. For the present study, the outcome should reflect aspects of psychosocial work environment factors. Inclusion criteria stated that the study should be a primary, quantitative study, published in a peer-reviewed journal. Only English language publications dated from January 2010 to February 2021 were included. Hence, any systematic reviews, meta-analyses, qualitative studies, theoretical articles, books or book chapters, short communications, editorials, purely descriptive studies, and dissertations were excluded. Studies where the work from home exposure was not specified as TWFH were excluded. A full description of the search including mesh terms can be found in supplementary 1.

### Study selection

To preliminarily assess whether the selected and retrieved studies met qualifications, the articles’ titles and abstracts were screened independently by pairs of researchers using Covidence ® software. Disagreements on whether a study should be included for full text review were resolved by discussion between the two involved researchers. In cases where agreement was not reached, a third researcher was involved, carrying out an individual evaluation.

Publications selected through preliminary screening were read in their entirety by pairs of researchers, again via Covidence software. The screening software ensured blinding, so no researcher decision was visible before both researchers had made a decision. Furthermore, who was paired to screen studies was also determined by the software, and unknown by the researchers prior to making a decision. As before, disagreements on whether a study should be included in the systematic review were resolved via discussion.

### Data extraction

A pre-defined data extraction spreadsheet with clear instructions was utilized to facilitate data extraction. Involved researchers ensured that consensus was reached prior to data extraction. Variables extracted included, but were not limited to: (1) TWFH exposures — including instruments, (2) work environment-related outcomes—including instruments, (3) study design, (4) country of study, (5) population occupation, (6) sample size, (7) response rate, (8) attrition, (9) control variables (if applicable), (10) mediating and moderating variables (if applicable), and (11) main findings and results. Not all studies reported on all the desired information.

### Risk of bias and quality of evidence

To assess the quality of individual studies, the Newcastle–Ottawa Quality Assessment Scale (NOS) was utilized. The NOS tool was chosen as it has been developed specifically to assess the quality of non-randomized studies for the purpose of inclusion in systematic reviews or meta-analyses (Stang 2010; Wells et al.). NOS operates in a star, or point, system, where points are rewarded across three domains; the selection of the study groups, the comparability of the groups, and the ascertainment of either the exposure or outcome of interest. For the purpose of the present review, three different versions of the NOS were included, one for each type of study design (cross-sectional, cohort, and randomized controlled trials (RCT)). These non-validated versions of NOS were adapted to include a single point score for self-report survey, as this is the most common, and in many cases the most suitable, method for measuring psychosocial and work-related concepts (Rosário et al. 2016; Useche et al. 2019). Similarly, questions on exposure gathered via structured surveys were considered to be gathered via “structured interview”, which resulted in one point score on the scale. Furthermore, individual study ratings were assigned following NOS scores based on AHRQ standards, which includes thresholds so that scores can be categorized into either rated poor, fair, or good (see supplementary 2 for an overview). As with the selection of studies, rating conflicts were resolved via discussion between at least two of the researchers involved.

To assess the overall certainty of evidence, three researchers evaluated the Grading of Recommendations Assessment, Development and Evaluation (GRADE) (Andrews et al. 2013) facilitated by the GRADEpro® software. GRADE is an often used procedure to rate the quality of the joint scientific evidence (e.g., not the individual study’s analysis as in NOS, but rather the combined evidence of the analyses on the topic) in systematic reviews, initially developed to help form recommendations in clinical guidelines for evidence-based practice. Following GRADE, RCT studies are initially considered high-quality evidence, while observational studies are considered low-quality evidence. Subsequently, five factors and corresponding rating steps may result in rating up or down. Consequently, the quality of evidence of the combined studies receives one of the four scores: very low, low, moderate, or high (Schünemann et al. 2013).

### Data synthesis

The heterogeneities in both the definitions, designs and methods for measuring TWFH as well as the outcomes were judged to be extensive. Hence, we considered the primary studies unsuitable for quantitative pooling of data or meta-analyses. We carried out a narrative analysis of the included studies, where characteristics and summary of results are described for each study. Thereafter, we formed outcome categories by grouping each respective work environmental outcome together with other similar outcomes across studies. Each of these outcome categories was finally evaluated by GRADE to determine their overall certainty of evidence.

## Results

### Study selection

The initial search resulted in 2808 hits, while the repeated search resulted in 569 new hits, a total of 3377 hits. A total of 3354 references were reviewed after sorting for duplicates. Preliminary screening resulted in 289 publications being selected for further screening.

**Fig. 1:**
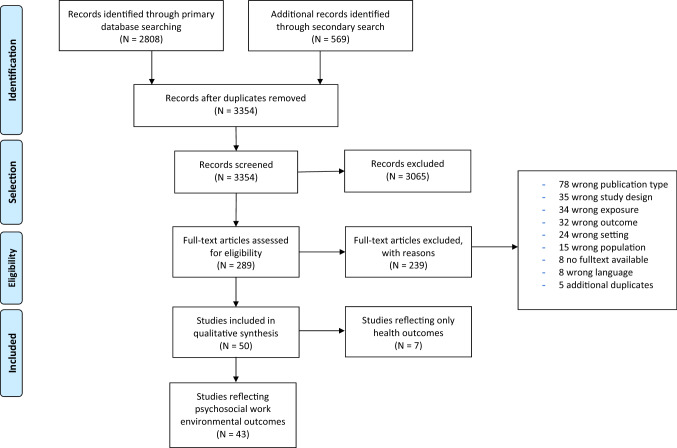
PRISMA flow diagram

Of the 289 publications read in their entirety by pairs of researchers, 50 publications met the selection criteria reflecting both outcome categories: work environment effects and employee health. Out of these 50 articles, 43 publications examined the relationship between work from home and one or more work environment factors. See Fig. 1 for an overview of the screening and selection process.

### Study descriptives

Of the 43 studies included in the present review, 30 had a cross-sectional design, 12 were longitudinal studies, and one a randomized controlled trial. Since several studies included more than one outcome, the number of analyses included in this review is higher than the number of individual studies, and the 43 studies included a total of 71 analyses (see Table 1). As such, the number of analyses included in this review is higher than the number of individual studies. Thus, based on the analyses in the individual studies, a total of 14 separate work environment categories were identified. Table 1 shows these specific categories, as well as how many and which type of analyses investigated these categories. Eighteen studies were conducted in the USA, five in the Netherlands, four in the UK, three in Germany, three in Belgium, two in Canada, and one in each of the countries Costa Rica, South Africa, Italy, China, Finland, and Australia. One study gathered evidence from several European countries (Bulgaria, Finland, Germany, Hungary, the Netherlands, Portugal, Spain, Sweden, and the UK), while another just specified Europe in general as they used data provided by the sixth European Working Conditions Survey. Sample sizes ranged from 61 to 376,577. An overview on study characteristics can be found in Table 2.

**Table 1:** Outcome categories with respective number and types of analyses

Outcome category	Cross-sectional	Cohort	RCT
Work–life balance	12	3	0
Job satisfaction	9	3	1
Productivity	7	3	1
Turnover intention	3	3	1
Working hours	4	1	1
Work engagement	3	2	0
Organizational commitment	4	1	0
Decision latitude	2	0	0
Self-leadership	2	0	0
Creativity	0	1	0
Professional isolation	0	1	0
Work concentration	0	1	0
Perceived fairness	1	0	0
Timing of work	1	0	0

**Table 2:** Study characteristics and summary of study results

Author	Title	Summary of findings	Exposure	Outcome	Outcome category	Type of work	Country	*N*	Quality	Design
Baard and Thomas (2010)	Teleworking in South Africa: employee benefits and challenges	TWFH was associated with an increased work–life balance and less stress, but also an increase in working hours Findings: Increased working hours Increased work–life balance	Type of telework	Experienced benefits and challenges with telework	Work-life balance, Working hours	Finance and tele-communication	South Africa	63	Poor	Cross-sectional
Higgins et al. (2014)	The relationship between work arrangements and work-family conflict	TWFH were associated with increased conflict between work and family life Findings: Decreased work–life balance	Ordinary working days, compressed working weeks, flexi-time or telework	Work interferes with family (WFC) and family interferes with work (FWC)	Work-life balance	General working population (with dependent care responsibilities)	Canada	16,145	Good	Cross-sectional
van der Lippe and Lippényi (2020)	Beyond formal access: organizational context, working from home, and work–family conflict of men and women in European workplaces	TWFH was associated with increased conflict between work and family life Findings: Decreased work–life balance	WFH	Work–family conflict	Work-life balance	Industry, higher education, health care, IT, transport, logistics and finance	Bulgaria, Finland, Germany, Hungary, Netherlands, Portugal, Spain, Sweden, UK	11,011	Good	Cross-sectional
Morganson et al. (2010)	Comparing telework locations and traditional work arrangements: differences in work–life balance support, job satisfaction, and inclusion	TWFH was associated with a decreased experience of inclusion in the workplace, but was not found to affect the balance between work or job satisfaction Findings: Null-findings work–balance Null-finding job satisfaction Decreased org. com	Workplace (main office, client location, satellite office, and home)	Balance between work and family, social support, job satisfaction and inclusion	Work-life balance, Organizational commitment Job satisfaction	Non-profit engineering company	USA	578	Good	Cross-sectional
Palumbo (2020)	Let me go to the office! An investigation into the side effects of working from home on work–life balance	TWFH was associated with increased conflict between work and family life Findings: Decreased work–life balance	Home-based telecommuting	Work-family balance	Work-life balance	Public servants	Europe	9877	Poor	Cross-sectional
Sarbu (2018)	The role of telecommuting for work-family conflict among German employees	TWFH was associated with an increased degree of conflict between work and family life. Increased degree of WFH was also associated with increasing conflict Findings: Decreased work–life balance	Telecommuting	Work-family conflict	Work-life balance	General working population	Germany	15,035	Good	Cross-sectional
Solis (2016)	Telework: conditions that have a positive and negative impact on the work-family conflict	Increased number of days and duration of TWFH was associated with increased conflict between work and family life Findings: Decreased work–life balance	Amount of TWFH	Work–family conflict	Work-life balance	Teleworkers in public institutions	Costa Rica	142	Poor	Cross-sectional
Dockery and Bawa (2018)	When two worlds collude: working from home and family functioning in Australia	TWFH was associated with less work-family conflict Findings: Increased work–life balance	TWFH	Family functioning	Work-life balance	General working population	Australia	56,013	Good	Cross-sectional
Felstead and Henseke (2017)	Assessing the growth of remote working and its consequences for effort, well-being and work–life balance	TWFH was associated with increased job satisfaction, organizational affiliation and effort, but at the same time increased conflict between work and family life Findings: Decreased work–life balance Increased job satisfaction Increased org. com Increased productivity	TWFH	Self-reported work effort, organizational commitment, job satisfaction, and balance between work and family	Work-life balance, Productivity, organizational commitment, job satisfaction	General working population	UK	14,457	Good	Cross-sectional
Eng et al. (2010)	What influences work-family conflict? The function of work support and working from home	Degree of TWFH was not found to affect the balance between employees’ work and family life Findings: Null-findings work–life balance	TWFH	Work interferes with family (WFC) and family interferes with work (FWC)	Work-life balance	Large US company	USA	1103	Poor	Cross-sectional
Restrepo and Zeballos (2020)	The effect of working from home on major time allocations with a focus on food-related activities	TWFH was associated with fewer active working hours and increased time spent on leisure activities Findings: Increased work–life balance Decreased working hours	TWFH	Spending time at work and in leisure time	Work-life balance, working hours	Administrative jobs	USA	2441	Fair	Cross-sectional
Duxbury and Halinski (2014)	When more is less: an examination of the relationship between hours in telework and role overload	The amount of TWFH was associated with increased conflict between work and family life Findings: Decreased work–life balance	TWFH	Work role overload, family role overload	Work-life balance	Employees in knowledge companies	Canada	1806	Poor	Cross-sectional
Bae and Kim (2016)	The impact of decoupling of telework on job satisfaction in U.S. federal agencies: does gender matter?	Employees in companies with the opportunity to TWFH reported more job satisfaction than employees in companies that did not allow WFH Findings: Increased job satisfaction	Employee participation in telework	Job satisfaction	Job satisfaction	US federal government agency	USA	219,450	Good	Cross-sectional
Caillier (2011b)	The impact of teleworking on work motivation in a U.S. federal government agency	Teleworkers (infrequent and frequent) did not consistently have higher levels of job satisfaction, organizational commitment and job involvement than non-teleworkers Findings: Null-finding job satisfaction Null-finding job engagement Null-finding org.com	Telework and the cause of telework	Job satisfaction, organizational commitment, job involvement	Job satisfaction, Work engagement, organizational commitment	US federal government agency	USA	263,475	Good	Cross-sectional
Caillier (2014)	Do role clarity and job satisfaction mediate the relationship between telework and work effort?	TWFH was associated with lower self-reported productivity, but increased job satisfaction Findings: Increased job satisfaction Decreased productivity	TWFH more than two days per week	Work effort, job satisfaction	Job satisfaction, productivity	US federal government agency	USA	266,376	Good	Cross-sectional
De Menezes and Kelliher (2017)	Flexible working, individual performance, and employee attitudes: comparing formal and informal arrangements	Opportunity for TWFH was associated with increased job satisfaction, stronger organizational commitment and increased productivity Findings: Increased org.com Increased job satisfaction Increased productivity	Informal and formal flexible work arrangements	Individual productivity, organizational commitment, job satisfaction	Job satisfaction, productivity, organizational commitment	International companies in pharmacy and finance	UK	2617	Poor	Cross-sectional
Fonner and Roloff (2010)	Why teleworkers are more satisfied with their jobs than are office-based workers: when less contact is beneficial	TWFH was associated with more job satisfaction Findings: Increased job satisfaction	Work arrangement	Job satisfaction	Job satisfaction	Administrative staff, both private and public sector	USA	192	Poor	Cross-sectional
Lee and Kim (2017)	A quasi-experimental examination of telework eligibility and participation in the U.S. federal government	Employees with the opportunity to TWFH reported increased job satisfaction, experience of a fairer workplace and lower intention to quit as compared to employees who did not have the opportunity to TWFH Findings: Increased job satisfaction Increased perceived fairness Decreased turnover intention	Opportunity and amount TWFH	Job satisfaction, perceived fairness and turnover intention	Job satisfaction, turnover intention, perceived fairness	US federal government agency	USA	Not included	Good	Cross-sectional
Gajendran et al. (2014)	Are telecommuters remotely good citizens? unpacking telecommuting's effects on performance via I‐deals and job resources	TWFH was associated with increased productivity and autonomy Findings: Increased productivity Increased autonomy	Type of telework	Task performance, contextual performance, perceived autonomy	Productivity,	Private and public sector companies	USA	466	Poor	Cross-sectional
Giménez-Nadal et al. (2019)	Work time and well-being for workers at home: evidence from the American time use survey	TWFH was associated with less active working hours and less work in core time compared to working days in the office Findings: Decreased work in core time Decreased working hours	TWFH	Working hours – timing of work	Working hours, timing of work	General working population	USA	5401	Fair	Cross-sectional
Neirotti et al. (2012)	Telework configurations and labor productivity: Some stylized facts	Home-based telework was not significantly associated with productivity Findings: Null-finding productivity	Telework configurations	Productivity	Productivity	Industry, trade, transport, logistics and IT	Italy	1134	Good	Cross-sectional
Peters et al. (2014)	Enjoying new ways to work: an HRM-process approach to study flow	Employees who teleworked from home ≥ 1d per week reported an increased workflow compared to employees who only worked in offices Findings: Increased productivity	TWFH	Work flow	Productivity	Private and public sector companies	The Netherlands	1114	Good	Cross-sectional
Caillier (2011a)	Are teleworkers less likely to report leave intentions in the United States federal government than non-teleworkers are?	Not having the opportunity for partial work from home was linked to a higher degree of intention to quit Findings: Decreased turnover intention	TWFH	Turnover intention	Turnover intention	US federal government agency	USA	263,475	Good	Cross-sectional
Choi (2017)	Managing flexible work arrangements in government: testing the effects of institutional and managerial support	Opportunity to choose WFH was associated with a lower intention to quit the job Employees who did not have the opportunity / were not allowed to work from home had the highest degree of intention to quit their jobs Findings: Less turnover intention	TWFH	Turnover intention	Turnover intention	US federal government agency	USA	376,577	Good	Cross-sectional
Sardeshmukh et al. (2012)	The impact of telework on exhaustion and job engagement: a job demands job resources model	TWFH was associated with lower job engagement the more they worked from home Findings: Decreased job engagement	Extent of TWFH	Work engagement	Work engagement	Logistics	USA	417	Poor	Cross-sectional
Vander Elst et al. (2017)	Not the extent of telecommuting, but job characteristics as proximal predictors for work-related well-being	The amount of teleworking from home was not associated with changes in job engagement, perceived self-leadership or control over decisions in one's own work Findings: Null-findings job engagement Null-findings self-leadership Null-findings decision latitude	Extent of telecommuting	Cynicism, work engagement, decision control	Work engagement, self-leadership, decision latitude	IT and communication	Belgium	878	Good	Cross-sectional
Possenriede et al. (2016)	Does temporal and locational flexibility of work increase the supply of working hours? Evidence from the Netherlands	Tele-home-work associated with increased actual working hours, but not contracted and preferred working hours Findings: Increased actual working hours	Flexi-time and tele-homework arrangements	Number of actual working hours, agreed working hours, preferred working hours	Working hours	General working population	The Netherlands	7164	Good	Cross-sectional
Chen and McDonald (2014)	Do networked workers have more control? the implications of teamwork, telework, ICTs, and social capital for job decision latitude	TWFH was associated with increased job decision latitude Findings: Increased decision latitude	TWFH	Decision latitude	Decision latitude	General working population	USA	1000	Poor	Cross-sectional
Müller and Niessen (2019)	Self-leadership in the context of part-time teleworking	On days of work from home, employees reported an increased degree of self-leadership compared to working days in the office Findings: Increased job satisfaction Increased self-leadership	Work location	Self-leadership, exhaustion, job satisfaction	Self-leadership, Job satisfaction	IT and communication, industry, health, finance, insurance, logistics	Germany	195	Good	Cross-sectional
Golden and Gajendran (2019)	Unpacking the Role of a Telecommuter’s Job in Their Performance: examining job complexity, problem solving, interdependence, and social support	Extent of TWFH was associated with increased job performance. Most distinctively for employees with complex jobs, jobs with low interdependence, jobs with low social support Findings: Increased productivity	Extent of TWFH	Job performance	Productivity	Marketing, IT, finance, sales	USA	273	Poor	Cross-sectional
Biron and van Veldhoven (2016)	When control becomes a liability rather than an asset: comparing home and office days among part-time teleworkers	Employees who worked partly from home reported a higher degree of concentration after working days in the office Findings: Increased concentration	TWFH	Work concentration	Work concentration	General working population	The Netherlands	77	Good	Longitudinal
Delanoeije et al. (2019)	Boundary role transitions: a day-to-day approach to explain the effects of home-based telework on work-to-home conflict and home-to-work conflict	Days working from home were associated with more conflicts at home, compared to working days in the office Findings: Decreased work–life balance	TWFH	Work-to-home conflict, home-to-work conflict	Work-life balance	Administrative jobs	Belgium	81	Poor	Longitudinal
Lapierre et al. (2016)	Juggling work and family responsibilities when involuntarily working more from home: a multi-wave study of financial sales professionals	Involuntarily working more from home was associated with higher strain-based WFC but not higher time-based WFC Findings: Decreased work–life balance	Involuntary TWFH	Strain-based WFC, time-based WFC	Work-life balance	Sales and advertising	The Netherlands	251	Good	Longitudinal
Kröll and Nüesch (2019)	The effects of flexible work practices on employee attitudes: evidence from a large-scale panel study in Germany	TWFH was associated with increased job satisfaction decreased turnover intention Findings: Increased job satisfaction Decreased turnover intention	Flexible work practices	Job satisfaction, turnover intention	Job satisfaction, turnover intention	General working population	Germany	22,042	Good	Longitudinal
Reuschke (2019)	The subjective well-being of homeworkers across life domains	TWFH was associated with increased job satisfaction Findings: Increased job satisfaction	TWFH	Satisfaction (differing aspects)	Job satisfaction	General working population	UK	3738	Good	Longitudinal
Vega et al. (2014)	A within-person examination of the effects of telework	Workers reported higher levels of productivity, job satisfaction og creativity when teleworking from home Findings: Increased job satisfaction Increased productivity Increased creativity	TWFH	Productivity, creativity, job satisfaction	Productivity, creativity, job satisfaction	US federal government agency	USA	180	Fair	Longitudinal
de Vries et al. (2018)	The benefits of teleworking in the public sector: reality or rhetoric?	WFH was associated with increased professional isolation and less organizational commitment, but no change in work commitment. Frequent contact between manager and employees (LMX) was found to reduce the experience of professional isolation Findings: Null-findings job engagement Decreased org.com Increased professional isolation NB!—moderation – LMX on isolation	TWFH	Organizational commitment, work commitment, professional isolation	Work engagement, organizational commitment, professional isolation	Public sector	The Netherlands	61	Poor	Longitudinal
Caillier (2017)	Do flexible work schedules reduce turnover intention in U.S. federal agencies?	TWFH was not associated with turnover intention Findings: Null-findings turnover intention	TWFH	Turnover intention	Turnover intention	US federal government agency	USA	376,577	Good	Longitudinal
Choi (2019)	Flexible work arrangements and employee retention: a longitudinal analysis of the federal workforces	TWFH was associated with a lower intention to quit the job Findings: Decreased turnover intention	Workers with the opportunity for TWFH	Voluntary turnover intention	Turnover intention	Public sector employees	USA	428	Good	Longitudinal
Nätti et al. (2011)	Work at home and time use in Finland	Employees who worked from home reported more working hours compared to employees in the office Findings: Increased productivity	Home-based work	Number of hours worked	Working hours	General working population	Finland	4587	Good	Longitudinal
Giovanis (2018)	The relationship between flexible employment arrangements and workplace performance in Great Britain	TWFH was associated with increased productivity Findings: Increased productivity	TWFH; flexible timing, compressed workweek	Workplace performance	Productivity	Sample of British workplaces with at least 5 employees	UK	N/A	Fair	Longitudinal
Delanoeije and Verbruggen (2020)	Between-persons and within-person effects of telework: a quasi-field experiment	TWFH was associated with increased work engagement and productivity, but also more conflict between work and leisure compared to working days in the office Findings: Decreased work–life balance Increased productivity Increased work engagement	TWFH two days a week	Work-to home conflict, work engagement, productivity	Work-life balance, productivity, Work engagement	Large international construction and property development firm	Belgium	64	Good	Longitudinal
Bloom et al. (2013)	Does working from home work? Evidence from a Chinese experiment	TWFH was associated with increased satisfaction and productivity, and more working minutes per shift and lower turnover intention Findings: Increased job satisfaction Increased productivity decreased turnover Increased working hours	TWFH	Turnover intention, job satisfaction, minutes worked, calls per minute	Productivity, Job satisfaction, turnover intention, Working hours	Travel agent	China	249	Poor	RCT

### Findings for psychosocial work environment factors

While some of the categories, such as job satisfaction, turnover intention, or productivity, occasionally are considered a consequence of the psychosocial work environment, rather than a self-standing work environment factor, we chose to include and categorize them as psychosocial work environment factors for the purpose of capturing the effects of TWFH on the psychosocial work environment in the broadest sense.

Individual study characteristics, results and NOS scores for each study are reported separately in Table 2.

#### Work–life balance

Work–life balance entails how workers manage the interface between time spent at and outside of work (Grzywacz and Butler 2007). Some studies included in this outcome category looked at work–life balance, a positive concept, while other studies looked at work–life conflict, which is a negative concept. Seven of the 12 cross-sectional analyses showed that TWFH was associated with a poorer balance between work and family life (Duxbury and Halinski 2014; Felstead and Henseke 2017; Higgins et al. 2014; Palumbo 2020; Sarbu 2018; Solis 2016; van der Lippe and Lippényi 2020), whereas 3 found that TWFH was associated with a better balance between work and family life (Baard and Thomas 2010; Dockery and Bawa 2018; Restrepo and Zeballos 2020), and 2 found no correlation or conflicting results (Eng et al. 2010; Morganson et al. 2010). Three out of three prospective analyses found that TWFH predicted poorer work–life balance (Delanoeije and Verbruggen 2020; Delanoeije et al. 2019; Lapierre et al. 2016). The overall quality of evidence for the relationship between TWFH and work–life balance was considered very low according to GRADE.

#### Job satisfaction

Seven out of the nine cross-sectional analyses suggested TWFH to be associated with higher job satisfaction (Bae and Kim 2016; Caillier 2014; De Menezes and Kelliher 2017; Felstead and Henseke 2017; Fonner and Roloff 2010; Lee and Kim 2017; Müller and Niessen 2019). Two of the cross-sectional analyses did not find associations or found contradicting results (Caillier 2011b; Morganson et al. 2010) The three prospective analyses suggested TWFH may lead to increased job satisfaction (Kröll and Nüesch 2019; Reuschke 2019; Vega et al. 2014). Finally, the one RCT investigating the effects of TWFH on job satisfaction also indicated a positive effect of TWFH on job satisfaction (Bloom et al. 2013). The assessment of the overall degree of evidence based on GRADE indicated low quality of evidence for the relationship between TWFH and job satisfaction.

#### Productivity

Productivity was defined in different ways in the studies included in this review, where some studies looked at labor performance, or work effort, others at financial performance.

Five of the cross-sectional analyses suggested associations between TWFH and higher productivity or better performance (De Menezes and Kelliher 2017; Felstead and Henseke 2017; Gajendran et al. 2014; Golden and Gajendran 2019; Peters et al. 2014), whereas the last two cross-sectional analyses found that TWFH was not associated (Neirotti et al. 2012) or associated with lower productivity (Caillier 2014). Three prospective analyses also suggested that TWFH resulted in increased productivity or better performance (Delanoeije and Verbruggen 2020; Giovanis 2018; Vega et al. 2014). Lastly, the one RCT included showed that those engaged in TWFH were more productive than those who worked from the employer’s premises (Bloom et al. 2013). GRADE scoring indicated low quality of evidence for a relationship between TWFH and productivity.

#### Turnover intention

Turnover intention reflects employee’s intention to leave or quit their current position. All three cross-sectional analyses found that TWFH was associated with lower turnover intention (Caillier 2011a; Choi 2017; Lee and Kim 2017). Furthermore, two of the prospective analyses also suggested TWFH resulted in lower turnover intention (Choi 2019; Kröll and Nüesch 2019). The last prospective analysis found no association (Caillier 2017). One analysis based on a RCT study showed lower turnover intention with TWFH (Bloom et al. 2013). The overall degree of evidence based on GRADE indicated low quality of evidence for TWFH and turnover intention.

#### Working hours

Out of the four cross-sectional analyses investigating TWFH and working hours, two found TWFH to be associated with fewer active working hours among those who TWFH (Giménez-Nadal et al. 2019; Restrepo and Zeballos 2020), while the other two suggested an association between TWFH and increased working hours (Baard and Thomas 2010; Possenriede et al. 2016). Both the longitudinal analysis (Nätti et al. 2011) and the analysis from the RCT study (Bloom et al. 2013) showed that TWFH was associated with an increase in working hours. GRADE indicated very low quality of evidence for TWFH and working hours.

#### Work engagement

Work engagement can be considered “the harnessing of organization member’s selves to their work roles: in engagement, people employ and express themselves physically, cognitively, emotionally and mentally during role performances” (Kahn 1990). One of the three cross-sectional analyses found that TWFH was associated with lower levels of work engagement (Sardeshmukh et al. 2012). The two remaining cross-sectional analyses found no association or contradicting results (Caillier 2011b; Vander Elst et al. 2017). One out of two prospective analysis found no relationship between TWFH and work engagement (de Vries et al. 2018) while the other suggested increased work engagement among employees who worked from home (Delanoeije and Verbruggen 2020). GRADE scoring indicated very low quality of evidence for the relationship between TWFH and work engagement.

#### Organizational commitment

A suggested definition for organizational commitment is “the relative strength of an individual’s identification with and involvement in a particular organization” (Mowday et al. 1979). Two out of the four cross-sectional analyses investigating TWFH and organizational commitment found a positive association (De Menezes and Kelliher 2017; Felstead and Henseke, 2017). One found TWFH to be associated with lower organizational commitment (Morganson et al. 2010), while the last cross-sectional analysis found no association (Caillier 2011b). The one prospective analysis showed TWFH to result in lower organizational commitment (de Vries et al. 2018). GRADE scoring indicated very low quality of evidence for the relationship between TWFH and organizational commitment.

#### Decision latitude

Decision latitude reflects the working individual's potential control over their tasks and their conduct during the working day (Karasek 1979). Two cross-sectional analyses investigated the relationship between TWFH and decision latitude, with one of the analyses indicating a positive relationship (Chen and McDonald 2014) and the other found no association (Vander Elst et al. 2017). GRADE scoring suggests very low quality of evidence for the relationship between TWFH and decision latitude.

#### Self-leadership

Self-leadership can be defined as using a specific set of behavioral and cognitive strategies to lead oneself (Neck and Houghton 2006). One of the cross-sectional analyses observed a positive association between TWFH and self-leadership (Müller and Niessen 2019), while the other did not find a significant association (Vander Elst et al. 2017). GRADE scoring suggests low quality of evidence for the relationship between TWFH and self-leadership.

#### Creativity

A single prospective analysis looked at effects of TWFH on employee creativity (Vega et al. 2014). This analysis found that employees may perform better on objective creative tasks when TWFH. The overall body of evidence following GRADE is rated very low.

#### Professional isolation

Professional isolation may be defined as an employee’s belief that he/she is disconnected with others in the workplace. In essence, professional isolation reflects the belief that one lacks sufficient connection to critical networks of influence and social contact (Diekema 1992; Miller, 1975). One prospective analysis showed that working either fully or partially from home may increase professional isolation (de Vries et al. 2018). The overall body of evidence for the association between TWFH and professional isolation following GRADE scoring is very low.

#### Work concentration

Only a single prospective analysis investigated how TWFH may affect work concentration (Biron and van Veldhoven 2016). This study found that part-time teleworkers experienced higher levels of work concentration. The overall body of evidence for the association between TWFH and work concentration following GRADE scoring is very low.

#### Perceived fairness

An employee’s perception of whether procedures and practices within the organization are just and fair define the employees’ level of perceived fairness (McFarlin and Sweeney 1992). A single cross-sectional analysis looked at the effects of TWFH on perceived fairness, and found TWFH to be associated with higher levels of perceived fairness (Lee and Kim 2017). The overall body of evidence following GRADE is rated very low.

#### Timing of work

Only one cross-sectional analysis reflected timing of work in people TWFH versus colleagues working at the office, and it found that workers TWFH spent less time working in traditional core working hours than their counterparts at the office (Giménez-Nadal et al. 2019). GRADE scoring indicated very low quality of evidence for the relationship between TWFH and timing of work.

## Discussion

The quality of evidence of effects on the fourteen suggested outcome categories ranged from very low to low, which suggests a lack of high-quality research, and that little can be concluded with regards to evidence on either positive or negative effects on TWFH on psychosocial work environment factors.

The present systematic review indicates that teleworking partially from home has a positive effect on work engagement, while working fully from home may have negative effects on work engagement. Several of the studies included the present systematic review seem to reflect on the need for freedom and flexibility in TWFH for these type arrangements to have positive effects on the experienced work environment. For example, turnover intention, where the turnover intentions of non-teleworkers are significantly different from those TWFH depending on one’s ability to choose, meaning that as long as working from home is voluntary and by choice, it may decrease employee’s turnover intention. Choi (2017) suggests that employees who were not offered a flexible work arrangement, i.e., did not have the freedom of choice to TWFH, reported the highest level of dissatisfaction with their work, while those employees who were eligible to work from home but decided not to make use of this arrangement reported the lowest turnover intention. This suggests that freedom of choice in TWFH may be of importance for possible positive effects. Similarly, Caillier (2011a) suggests that not having the opportunity to work from home was associated with a higher intention to quit.

Autonomy may therefore be a key factor in whether TWFH has negative or positive effects on the experienced psychosocial work environment. Technologies that allow TWFH empower employees as they create flexibility in when and where to engage in work, however the same technologies eliminate personal freedoms by increasing job demands and availability expectancies and blurring the boundary between work and private life. As mentioned before, this duality is sometimes referred to as the “empowerment/enslavement paradox” (Cohen et al. 2021; Jarvenpaa et al. 2005). A meta-analysis looking at 46 studies on the effects of telecommuting concluded that the investigated types of remote working had an overall positive effect on proximal and long-term outcomes, including work–life balance, job satisfaction, performance, turnover intentions and role stress, where these beneficial effects seemed to be at least partially mediated by employee autonomy (Gajendran and Harrison 2007).

The importance of flexibility in- and autonomy over ones work–life, including when to engage in TWFH, may explain when TWFH has beneficial effects. To suggest TWFH guidelines or recommendations, there is a need for high-quality research within this field. Furthermore, when reflecting on implementing TWFH arrangements, employers should keep in mind the need for flexibility and autonomy to reap potential TWFH benefits.

### Strengths and limitations

To our knowledge, this is the only recent systematic review evaluating the existing evidence on the effects of TWFH on the psychosocial work environment. Having quality up-to-date research available on the topic may be relevant for policy-makers and employers when evaluating TWFH arrangements and/or interventions, as such and other flexible working solutions may play in important role in future of working life. This systematic review was carried out following recommended guidelines and standards for planning, execution, and reporting, and may therefore be considered a quality document when referring to existing evidence on the relationship between TWFH and the psychosocial work environment.

The studies included in the current review were mostly cross-sectional, implying that no causal effects can be established. As such, it may be that TWFH and work environment factors are a consequence of a common denominator. For example, several studies report that employees in home offices are more satisfied with their work than those who only work from the office. However, if the study does not incorporate a reflection on causality, where when said employees first started TWFH and then reported increased satisfaction, an equally plausible explanation may be TWFH is more prevalent in jobs that are generally characterized by freedom and autonomy, and thus these employees are more satisfied with their job than workers who are restricted to work from office premises.

Furthermore, most studies were conducted in the USA, which means that findings may not be generalizable to other nations working environment, as work and work environment factors, including e.g., organizational culture, occupational health regulations, and working arrangements differ greatly.

Mechanisms explaining how TWFH may influence the psychosocial work environment are lacking, and since most studies only reflect one or a few work environment factors, interrelations between factors, as well as potential moderating, mediating, or reciprocal effects are underexplored. Furthermore, the work environment outcomes that have been explored in the included studies do not discuss a number of factors that previous research has shown are of importance for the work environment and productivity, such as role conflict and role clarity, leadership, or social support from colleagues and managers (Wännström et al. 2009).

While all studies included reflected TWFH, this is not a homogeneous construct and may cover differing work situations, such as freedom and flexibility over TWFH, number of hours worked from home, task performed, or job type. This may also limit the generalizability of findings. Moreover, there was significant variation in how several of the work environment factors were defined between the included studies.

## Conclusion

This review provides an overview of the existing research on the relationship between telework from home and the psychosocial work environment. This study reveals that there is a lack of high-quality research investigating effects of TWFH on the psychosocial work environment, making it difficult to make clear evidence-based decisions. Most of the included studies had a cross-sectional design, making it impossible to conclude on causality. For many office occupations, it would be beneficial and likely feasible to use randomized controlled designs or other types of intervention designs with a reasonable follow-up. Such designs would contribute to a higher quality of knowledge on the effect of TWFH on the psychosocial work environment. We also believe that improvement in quality could be achieved by more detailed localisation of where the remote work is carried out and by precise measures of time spent TWFH. One solution could be to sample working hours by objective measures such as electronic time stamps, since this possibly would reduce bias such recall bias. Another improvement would be the investigation of factors that previous research has shown are of importance for the work environment. Such knowledge is crucial to provide future guidelines and recommendations for the use of TWFH regarding the psychosocial work environment.

## Supplementary Information

Below is the link to the electronic supplementary material.

Supplementary file1 (PDF 93 KB)

Supplementary file2 (DOCX 19 KB)

## Data Availability

All relevant data are included in the manuscript or supplementary.
