# Supplementary material for: The relationship between telework from home and the psychosocial work environment: a systematic review
Source: Int Arch Occup Environ Health. 2022 Jul 13;95(10):2025–51. doi: 10.1007/s00420-022-01901-4 (PMC9652268; doi:10.1007/s00420-022-01901-4)
Supplement: Supplementary file 2 — Supplementary file2 (DOCX 19 KB) [file 420_2022_1901_MOESM2_ESM.docx]

**Supplementary 2: Thresholds for converting the NOS scales to AHRQ standards (good, fair, and poor):**

**Good quality:** 3 or 4 stars in the selection domain AND 1 or 2 stars in comparability domain AND 2 or 3 stars in the outcome/exposure domain

**Fair quality:** 2 stars in the selection domain AND 1 or 2 stars in comparability domain AND 2 or 3 stars in the outcome/exposure domain

**Poor quality:** 0 or 1 star in the selection domain OR 0 stars in comparability domain OR 0 or 1 star in the outcome/exposure domain
